# Associations between alcohol consumption and cardiovascular disease among long-term survivors of colorectal cancer: a population-based, retrospective cohort study

**DOI:** 10.1186/s12885-021-08436-w

**Published:** 2021-06-16

**Authors:** Gyeongsil Lee, Seogsong Jeong, Seulggie Choi, Kyae Hyung Kim, Jooyoung Chang, Seong Rae Kim, Kyuwoong Kim, Joung Sik Son, Sung Min Kim, Daein Choi, Sang Min Park

**Affiliations:** 1 Department of Family Medicine, Seoul National University Hospital, 101 Daehak-ro, Jongno-gu, Seoul, 03080 South Korea; 2 Department of Biomedical Sciences, Seoul National University Graduate School, 101 Daehak-ro, Jongno-gu, Seoul, 03080 South Korea; 3 Department of Medicine, Seoul National University College of Medicine, 101 Daehak-ro, Jongno-gu, Seoul, 03080 South Korea; 4 National Cancer Control Institute, National Cancer Center, Gyeonggi-do, Goyang-si, 10408 South Korea; 5 Department of Medicine, Mount Sinai Beth Israel, Icahn School of Medicine at Mount Sinai, New York, NY 10029 USA

**Keywords:** Colorectal cancer, Alcohol consumption, Cardiovascular disease

## Abstract

**Background:**

There is no evidence whether it is best to stop drinking alcohol at all or whether it is okay to drink a little in that light-to-moderate alcohol use was associated with low cardiovascular disease (CVD) compared to non-drinker among colorectal cancer (CRC) survivors, who are regarded as vulnerable to CVD. Therefore, we evaluated the association between alcohol consumption and incident CVD among long-term survivors of CRC.

**Methods:**

This population-based, retrospective cohort study utilized data from the Korean National Insurance Service of 20,653 long-term survivors of CRC diagnosed between 2006 and 2012. Participants were followed up to the date of CVD, death, or December 31, 2018. All patients were categorized according to their daily alcohol consumption (g/day). The outcomes were incident CVD, including ischemic heart disease (IHD) and ischemic and hemorrhagic stroke, analyzed using the Cox proportional hazards regression after adjusting for cardiovascular risk factors and history of chemotherapy and radiotherapy.

**Results:**

There was no association between alcohol consumption and incident CVD among long-term survivors of CRC. Additionally, hazardous alcohol consumption (≥ 40 g/day in male patients and ≥ 20 g/day in female patients) was associated with increased CVD, ischemic stroke, and hemorrhagic stroke (adjusted hazard ratio [95% confidence interval]: 1.51 [1.15–1.97], 1.60 [1.03–2.48], and 2.65 [1.25–5.62], respectively) compared with non-drinkers.

**Conclusion:**

No discernable protective association was found between alcohol consumption and incident CVD for even light-to-moderate drinking among long-term survivors of CRC. Alcohol consumption ≥40 g/day in male patients and ≥ 20 g/day in female patients was associated with an increased risk of stroke compared with non-drinkers. These novel results provide useful evidence when advising survivors of CRC regarding alcohol use.

**Supplementary Information:**

The online version contains supplementary material available at 10.1186/s12885-021-08436-w.

## Background

Colorectal cancer (CRC) is the third most diagnosed cancer worldwide [1] and the second among South Korean adults [2]. Although the global CRC mortality is still high, some countries, including the United States, United Kingdom, and South Korea, report high 5-year survival rates for CRC [3]. Early detection, progress in national cancer screening, and advances in therapeutics facilitated the decrease in cancer mortality that, in turn, increased active life expectancy [4]. Accordingly, long-term survivors of CRC are now faced with other health problems, including obesity, hypertension, diabetes, dyslipidemia, and cardiovascular disease (CVD).

Usually, 5 years after diagnosis and treatment of CRC, patients are informed of 5-year survival and recommended self-management with community care. For this reason, the lifestyle of long-term CRC survivors tends to return to the initial state before diagnosis, particularly alcohol habit. However, there is no evidence whether it is best to stop drinking alcohol at all or whether it is okay to drink a little in that light-to-moderate alcohol use was associated with low ischemic heart disease (IHD) and stroke mortality compared to non-drinker [5]. Therefore, we investigated the association between alcohol use and CVD among long-term survivors of CRC.

## Methods

### Study overview and patient population

The Korean National Health Insurance Service (NHIS) is a mandatory health insurance system for all citizens, including detailed data on demographics, lifestyle behaviors, outpatient department visits, hospitalizations, pharmacological prescriptions, surgical and adjuvant treatments, and laboratory examinations [6]. All citizens aged 40 years or older are eligible for a biannual screening that involves self-reported questionnaires regarding medical history, behavioral characteristics, anthropometric measurements, and laboratory findings [7]. In the Korean NHIS, critical codes for cancer were implemented in 2004. Therefore, patients with cancer are offered additional critical condition codes that are adopted only when a diagnosis of cancer is confirmed [8]. These codes provide reimbursement benefits for cancer-associated management costs based on the household incomes of patients. The diagnosis of CRC was made within the dataset by the attending physicians who recorded a primary diagnosis according to the International Classification of Diseases, Tenth Revision (ICD-10) codes.

This population-based, retrospective cohort study utilized data from the Korean NHIS. The inclusion criteria were patients with new-onset CRC who survived at least 5 years and had available demographic characteristics, follow-up information, and clinical data. Patients with CVD before the index date, defined as the date of 5-year survival for each participant, and those with missing values for covariates were excluded. All patients were followed up from the index date to the date of CVD, death, or December 31, 2018 (see Figure S1 in a supplementary file). This study was approved by the Institutional Review Board of Seoul National University (approval number E-2004-191-1119). The requirement for informed consent was waived by the review board, as the NHIS database is anonymized by strict confidentiality guidelines.

Among the 33,483 patients who were diagnosed with CRC (ICD-10 codes, C17–C21) and received health examinations, we excluded 5869 patients with CRC before 2006 to restrict the study population to only those patients with newly diagnosed CRC. To reconcile the starting point of observation and detect new-onset CVD, we excluded 5905 patients who had CVD prior to the index date. In addition, 1056 patients who had missing values for covariates were excluded. The final study population included 20,653 patients with CRC who survived ≥5 years after the initial diagnosis.

### Exposure, outcomes, and covariates

Information on alcohol consumption was collected and included weekly drinking frequency (times per week) and daily consumption (standard drinks per day). One standard drink was defined as 10 g of alcohol. Alcohol-related classification followed the standards of the World Health Organization (WHO) [9]. A working definition from the WHO standards describes hazardous drinking as a regular, average alcohol consumption of ≥40 g/day for males and ≥ 20 g/day for females. Therefore, high alcohol consumption for male and female participants in this study was designated as ≥40 g/day and ≥ 20 g/day, respectively. CVD was the primary outcome and was defined as ≥2 days of hospitalization due to IHD or stroke. Before admission, the attending physician recorded a primary diagnosis according to the ICD-10 codes. The ICD-10 codes for CVD (I20–I25 and I60–I69), IHD (I20–I25), stroke (I60–I69), ischemic stroke (I63), and hemorrhagic stroke (I61–I62) were derived from the guidelines of the American Heart Association [10].

A self-reported questionnaire was used to obtain data on household income; history of dyslipidemia, hypertension, and diabetes mellitus; smoking status; alcohol consumption (as defined above); and physical activity. Physiological and serological measurements were collected at a health examination performed within 2 years prior to the 5-year survival date and included body mass index, waist circumference, blood pressure, fasting serum glucose, total cholesterol, and liver function tests. The Charlson Comorbidity Index (CCI) was calculated in accordance with a previous study [11]. Before hospital admission for a CVD event, the attending physician recorded a primary diagnosis using the ICD-10 codes for CVD as described above.

### Statistical analyses

Continuous and categorical variables are presented as the median (interquartile range) and number (%), respectively. The hazard ratio (HR) and 95% confidence interval (CI) for the risks of CVD, IHD, stroke, ischemic stroke, and hemorrhagic stroke were evaluated using Cox proportional hazards regression after adjusting for age (continuous: years); sex (categorical: male, female); household income (categorical: 4 quartiles); body mass index (continuous: kg/m^2^); systolic blood pressure (continuous: mmHg); fasting serum glucose (continuous: mg/dL); total cholesterol (continuous: mg/dL); smoking (categorical: never and ever); physical activity, including walking and moderate or vigorous physical activity (categorical: 0, 1–2, 3–4, and ≥ 5 days per week); CCI (continuous); history of chemotherapy (categorical: yes, no); and history of radiotherapy (categorical: yes, no). The proportional hazards assumption was tested graphically based on the scaled Schoenfeld residuals, and we detected no violation in the assumption for proportionality. Alcohol consumption was coded using a restricted cubic spline function with four knots located at the 5th, 33rd, 67th, and 95th percentiles based on the amount of alcohol intake presented using the penalized B-spline function. Patients with no alcohol consumption were set as the reference group. A *P* value of less than 0.05 was considered reflective of statistical significance. All statistical analyses and data collection were carried out using the SAS Enterprise Guide 7.1 (SAS Institute, Cary, NC, USA).

## Results

**Fig. 1:**
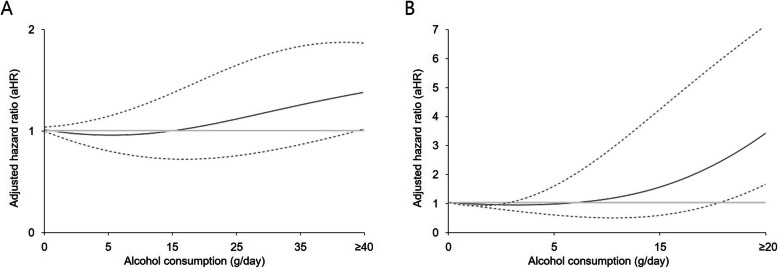
Restricted cubic spline used for evaluating the association between alcohol consumption and cardiovascular diseases. Alcohol consumption was coded using a restricted cubic spline function with four knots located at the 5th, 33rd, 67th, and 95th percentiles based on the amount of alcohol intake and was presented with the penalized B-spline function. The upper cut-offs for the amount of alcohol intake were ≥ 40 and ≥ 20 g/day for male and female participants, respectively. The confidence intervals are presented with dashed lines. Hazard ratios were calculated using Cox proportional hazards regression analysis after adjusting for age, household income, systolic blood pressure, body mass index, fasting serum glucose, total cholesterol, smoking, walking, moderate-to-vigorous physical activity, Charlson comorbidity index, history of chemotherapy, and history of radiotherapy. **a** Cardiovascular disease risk in male participants. **b** Cardiovascular disease risk in female participants

Table 1 shows the general characteristics of the study population according to sex. The proportion of non-drinkers was higher in female patients than in male patients. The median follow-up period was 3.2 years, and the longest follow-up period was 8.0 years. Figure 1 depicts the association of alcohol consumption with the risk of CVD. Alcohol consumption of ≥40 g/day in male patients and ≥ 20 g/day in female patients was associated with increased CVD compared with the non-drinking group after adjusting for the variables described above, whereas the lowest risks were found in the 10–19.9 g/day group for male patients and the < 5 g/day group for female patients. The quantitative values for the adjusted HR (aHR) for CVD, IHD, and stroke for male and female participants are presented in Tables 2 and 3, respectively.

**Table 1:** Baseline characteristics of the participants

Characteristic	Overall (*n* = 20,653)	Male (*n* = 11,774)	Female (*n* = 8879)
Age, years	64 (56–72)	65 (57–72)	63 (55–71)
Household income			
First quartile	3569 (17.3)	1938 (16.5)	1631 (18.4)
Second quartile	3455 (16.7)	1950 (16.6)	1505 (16.9)
Third quartile	5013 (24.3)	2811 (23.9)	2202 (24.8)
Fourth quartile	8616 (41.7)	5075 (43.1)	3541 (39.9)
Body mass index, kg/m^2^	23.6 (21.6–25.7)	23.8 (21.9–25.7)	23.3 (21.4–25.6)
Waist circumference, cm	82 (76–88)	84 (79–90)	78 (72–84)
Systolic blood pressure, mmHg	123 (113–133)	125 (115–135)	120 (110–131)
Diastolic blood pressure, mmHg	77 (70–80)	78 (70–81)	75 (69–80)
Fasting serum glucose, mg/dL	97 (89–108)	99 (90–111)	95 (88–105)
Total cholesterol, mg/dL	191 (168–217)	187 (163–212)	197 (174–224)
Aspartate aminotransferase, IU/L	24 (20–29)	25 (20–30)	23 (20–28)
Alanine aminotransferase, IU/L	20 (15–28)	22 (16–30)	18 (14–24)
γ–glutamyl transpeptidase, IU/L	23 (16–36)	27 (19–43)	18 (13–25)
Smoking status			
Never	12,818 (62.1)	4239 (36.0)	8579 (96.6)
Previous	5551 (26.9)	5393 (45.8)	158 (1.8)
Current	2284 (11.1)	2142 (18.2)	142 (1.6)
Alcohol consumption, day(s)/week			
0	14,972 (72.5)	6888 (58.5)	8084 (91.0)
1–2	3618 (17.5)	2955 (25.1)	663 (7.5)
3–4	1268 (6.1)	1178 (10.0)	90 (1.0)
≥ 5	795 (3.9)	753 (6.4)	42 (0.5)
Physical activity, day(s)/week			
0	5410 (26.2)	2967 (25.2)	2443 (27.5)
1–2	3519 (17.0)	2004 (17.0)	1515 (17.1)
3–4	4429 (21.4)	2448 (20.8)	1981 (22.3)
≥ 5	7295 (35.3)	4355 (37.0)	2940 (33.1)
Disease history			
Hypertension	5816 (28.2)	3398 (28.9)	2418 (27.2)
Diabetes mellitus	2494 (12.1)	1581 (13.4)	913 (10.3)
Dyslipidemia	1046 (5.1)	413 (3.5)	633 (7.1)
Charlson Comorbidity Index	3 (2–6)	3 (2–5)	4 (2–6)
History of chemotherapy	6354 (30.8)	3347 (28.4)	3007 (33.9)
History of radiotherapy	2874 (13.9)	1195 (10.2)	1679 (18.9)

Data are median (interquartile range) or n (%)

**Table 2:** Alcohol’s effects on CVD, IHD, and stroke risks among male long-term survivors of colorectal cancer

Outcome	0 g/day (*n* = 6888)	0.1–9.9 g/day (*n* = 1903)	10–19.9 g/day (*n* = 1064)	20–29.9 g/day (*n* = 781)	30–39.9 g/day (*n* = 350)	≥ 40 g/day (*n* = 788)	*P* for trend
CVD							0.148
Event (%)	369 (5.4)	92 (4.8)	47 (4.4)	43 (5.5)	21 (6.0)	59 (7.5)	
Person-years	22,818	6295	3525	2441	1120	2688	
HR (95% CI)	1.00 (Reference)	1.00 (0.80–1.27)	0.89 (0.65–1.21)	1.25 (0.90–1.73)	1.16 (0.74–1.82)	1.40 (1.05–1.86)	
IHD							0.840
Event (%)	162 (2.4)	46 (2.4)	21 (2.0)	20 (2.6)	7 (2.0)	21 (2.7)	
Person-years	23,305	6407	3578	2492	1151	2764	
HR (95% CI)	1.00 (Reference)	1.09 (0.78–1.52)	0.86 (0.54–1.36)	1.24 (0.77–2.01)	0.84 (0.39–1.80)	1.09 (0.69–1.75)	
Stroke							0.034
Event (%)	227 (3.3)	49 (2.6)	29 (2.7)	29 (3.7)	14 (4.0)	42 (5.3)	
Person-years	23,142	6408	3580	2477	1137	2738	
HR (95% CI)	1.00 (Reference)	0.89 (0.65–1.22)	0.92 (0.62–1.36)	1.41 (0.95–2.10)	1.28 (0.74–2.21)	1.61 (1.14–2.26)	

HR calculated using Cox proportional hazards regression after adjustments for age, household income, body mass index, systolic blood pressure, fasting serum glucose, total cholesterol, smoking, walking, moderate-to-vigorous physical activity, Charlson Comorbidity Index, history of chemotherapy, and history of radiotherapy

*CVD* cardiovascular disease, *IHD* ischemic heart disease, *HR* hazard ratio, *CI* confidence interval

**Table 3:** Alcohol’s effects on CVD, IHD, and stroke risks among female long-term survivors of colorectal cancer

Outcome	0 g/day (*n* = 8084)	0.1–9.9 g/day (*n* = 606)	10–19.9 g/day (*n* = 95)	≥ 20 g/day (*n* = 94)	*P* for trend
CVD					0.011
Event (%)	322 (4.0)	16 (2.6)	4 (4.2)	8 (8.5)	
Person-years	26,408	1905	299	287	
HR (95% CI)	1.00 (Reference)	0.95 (0.57–1.58)	1.54 (0.56–4.23)	3.40 (1.63–7.12)	
IHD					0.514
Event (%)	148 (1.8)	12 (1.7)^a^		1 (1.1)	
Person-years	26,831	2222		305	
HR (95% CI)	1.00 (Reference)	1.42 (0.77–2.59)		0.82 (0.11–5.96)	
Stroke					< 0.001
Event (%)	195 (2.4)	8 (1.1)^a^		7 (7.4)	
Person-years	26,757	2224		291	
HR (95% CI)	1.00 (Reference)	0.67 (0.33–1.38)		4.52 (2.02–10.14)	

HR calculated using Cox proportional hazards regression after adjustments for age, household income, body mass index, systolic blood pressure, fasting serum glucose, total cholesterol, smoking, physical activity, Charlson Comorbidity Index, history of chemotherapy, and history of radiotherapy

*CVD* cardiovascular disease, *IHD* ischemic heart disease, *HR* hazard ratio, *CI* confidence interval

^a^0.1–9.9 g/day and 10–19.9 g/day groups were merged due to the limited number of events

To confirm if the association between alcohol consumption and CVD risk was homogeneous regardless of patient characteristics among male participants, subgroup analyses were performed (see Table S1 in a supplementary file). There was no beneficial amount of drinking in any of the stratified subgroups. By contrast, consuming 10–19.9 g/day significantly worsened the prognosis of patients with dyslipidemia (aHR, 7.47; 95% CI, 1.90–29.37; *P* = 0.004) in terms of CVD. In subgroup analyses of female participants, alcohol consumption was not significantly associated with CVD (see Table S2 in a supplementary file). Female patients who consumed ≥20 g/day of alcohol maintained significant increases in CVD risk in most subgroups and 10–19.9 g/day was significantly harmful in patients with diabetes mellitus (aHR, 8.38; 95% CI, 1.62–43.22; *P* = 0.011).

When stratified according to the WHO criteria, moderate alcohol consumption (males, < 40 g/day; females, < 20 g/day) did not increase or decrease the risk for CVD, IHD, total stroke, ischemic stroke, and hemorrhagic stroke (Table 4). However, hazardous alcohol consumption (males, ≥ 40 g/day; females, ≥ 20 g/day) significantly elevated the risk for CVD, which was mainly attributed to stroke, including both ischemic and hemorrhagic stroke. The subgroup analyses by sex, age, body mass index, chronic disease, comorbidity, smoking, or physical activity demonstrated similar tendencies as those revealed in the main results (Table S3 in a supplementary file).

**Table 4:** Alcohol’s effects on CVD risks among CRC survivors according to the World Health Organization classification

Outcome	No drinking (*n* = 14,972)	Moderate drinking < 20 g/day for females and < 40 g/day for males (*n* = 4779)	Hazardous drinking ≥ 20 g/day for females and ≥ 40 g/day for males (*n* = 882)	*P* for trend
CVD				
Event (%)	691 (4.6)	223 (4.6)	67 (7.6)	
Person-years	49,226	15,585	2975	
HR (95% CI)	1.00 (Reference)	1.02 (0.88–1.19)	1.60 (1.25–2.06)	0.001
aHR (95% CI)^a^	1.00 (Reference)	1.06 (0.90–1.25)	1.51 (1.15–1.97)	0.010
IHD				
Event (%)	310 (2.1)	106 (2.2)	22 (2.5)	
Person-year	50,136	15,850	3069	
HR (95% CI)	1.00 (Reference)	1.08 (0.87–1.35)	1.16 (0.75–1.78)	0.661
aHR (95% CI)^a^	1.00 (Reference)	1.09 (0.85–1.39)	1.07 (0.68–1.68)	0.794
Stroke				
Event (%)	422 (2.8)	129 (2.7)	49 (5.6)	
Person-years	49,899	15,826	3030	
HR (95% CI)	1.00 (Reference)	0.97 (0.79–1.18)	1.91 (1.42–2.56)	< 0.001
aHR (95% CI)^a^	1.00 (Reference)	1.01 (0.82–1.26)	1.77 (1.29–2.42)	0.001
Ischemic stroke				
Event (%)	210 (1.4)	70 (1.5)	25 (2.8)	
Person-years	50,415	15,962	3095	
HR (95% CI)	1.00 (Reference)	1.06 (0.81–1.38)	1.93 (1.28–2.92)	0.017
aHR (95% CI)^a^	1.00 (Reference)	1.04 (0.77–1.39)	1.60 (1.03–2.48)	0.107
Hemorrhagic stroke				
Event (%)	45 (0.3)	15 (0.3)	10 (1.1)	
Person-years	50,783	16,075	3108	
HR (95% CI)	1.00 (Reference)	1.05 (0.59–1.88)	3.60 (1.82–7.15)	0.001
aHR (95% CI)^a^	1.00 (Reference)	0.87 (0.46–1.63)	2.65 (1.25–5.62)	0.017

HR calculated using Cox proportional hazards regression

*CVD* cardiovascular disease, *CRC* colorectal cancer, *HR* hazard ratio, *aHR* adjusted hazard ratio, *CI* confidence interval, *IHD* ischemic heart disease

^a^Adjusted for age, sex, household income, body mass index, systolic blood pressure, fasting serum glucose, total cholesterol, smoking, physical activity, Charlson Comorbidity Index, history of chemotherapy, and history of radiotherapy

## Discussion

In this large, retrospective cohort study, no discernable protective association was found between alcohol consumption and CVD incidence, even after light-to-moderate drinking, among long-term survivors of CRC. Alcohol consumption of ≥40 g/day in male patients and ≥ 20 g/day in female patients was associated with increased CVD, which was mainly attributed to stroke rather than IHD. To the best of our knowledge, this is the first study to investigate the association between alcohol consumption and CVD among long-term (minimum of 5 years) survivors of CRC. Our results may provide important and useful evidence when communicating with cancer survivors regarding alcohol use.

The protective effects of light-to-moderate alcohol consumption on the development of CVD have been extensively investigated, although mainly among healthy adults [5, 12]. The protective mechanisms were regarded as improving CV risk factors, such as increasing high-density lipoprotein cholesterol and improving insulin resistance [13, 14]; changing hemostatic factors, such as decreasing fibrinogen, platelet activation, and its aggregation [15]; and altering inflammation and oxidative stress [16]. However, we found that there were no protective effects of alcohol consumption on CVD developments among long-term survivors of CRC. Similar results were found when unadjusted or only adjusted for age and household income for each male and female long-term CRC survivors.

One recent study on the effects of moderate alcohol consumption provides clues as to why there was no favorable effect of light-to-moderate alcohol consumption in our study. This systematic review and meta-analysis reported that there were immediate toxic effects on the cardiovascular system following moderate alcohol consumption, but protective effects were observed after 24 h [17]. The acute changes included increased heart rate and blood pressure resulting from activation of the renin-angiotensin system, increased vascular reactivity, and inhibition of endothelial nitric oxide production [18]. Another study demonstrated that an acute, negative inotropic effect by alcohol in isolated myocardium resulted in weakened heart contractions [19].

Survivors of cancer per se are potentially vulnerable to CVD due to cardiotoxic cancer therapeutics, including chemotherapy and radiotherapy [20]. In particular, 5-fluorouracil is one of the most widely used chemotherapeutic agents for CRC and has been reported to induce cardiotoxicity and long-term cardiovascular sequelae [21]. Furthermore, survivors of cancer are more likely to have CVD risk factors than are individuals without cancer. One large, retrospective cohort study reported that cancer survivors had significantly higher weights and rates of hypertension and dyslipidemia than did non-cancer controls [22]. Another epidemiological study suggested that common CVD risk factors play more prominent roles in CVD development in cancer survivors than in non-cancer controls [23]. Accordingly, the vascular vulnerability of survivors of CRC may lead to a lower capacity to buffer the acute adverse effects of alcohol and may possibly aggravate the adverse effects of alcohol. In our study, we found that the incidence of both ischemic and hemorrhagic stroke was elevated in the hazardous alcohol consumption group than in the abstainer group among survivors of CRC, and the magnitude was higher for hemorrhagic than for ischemic stroke. Key risk factors for hemorrhagic stroke are hypertension and atherosclerosis in the small vessels of the brain [24]. Therefore, survivors of CRC are vulnerable not only to ischemic changes but also to rapidly increasing blood pressure, which could be induced by alcohol consumption. Further studies are needed to understand the effects of alcohol consumption on the cardio- and cerebrovascular systems of cancer survivors.

Several limitations of this study should be noted. First, hospitalization for more than 2 days for IHD or stroke was used to define CVD incidence (based on ICD-10 codes). This may have resulted in the underestimation of chronic IHD that required no hospitalization. However, the accuracy of ICD-10 codes for CVD is greater than 80% [25]. Second, the approach to defining alcohol consumption was challenging. We could not distinguish the types of alcoholic beverages consumed, e.g., beer, soju (Korean distilled beverage), wine, or makgeolli (traditional Korean alcohol). According to a report on the trends of total alcoholic beverage consumption in Korea [26], approximately 40.2% was beer, 31.0% was soju, 16.2% was makgeolli, and 4.4% was wine. Instead of differentiating alcoholic beverages, we tried to unify the amount of ethanol consumed (g/day) considering the average alcohol concentration and quantity of one drink of each alcohol type. Third, the follow-up period was a median of 3.2 years and may have been too short to determine an accurate incidence of CVD associated with alcohol consumption. Conversely, because CRC per se is a risk factor for CVD, early incident IHD or stroke during follow-up may have been related to the CRC experience and not alcohol consumption. Nevertheless, the findings from a sensitivity analysis of the main results, excluding participants diagnosed CVD with the first follow-up, were also in line with those from the main results. Fourth, CVD risks of CRC survivors who reduced or stopped drinking due to severe illness and life-long non-drinkers were not evaluated. However, considering that the study population comprised patients diagnosed with CRC, it may not significantly affect our results, which requires further studies for validation. In addition, the results of the subgroup analyses should be considered as information for future studies, for which caution should be taken when interpreting primary findings considering insufficient justification and evaluation supported by the interaction with alcohol consumption. Another important concern is the use of thrombocyte aggregation inhibitors or other anticoagulants and the presence of thrombocytopenia or myeloproliferative disease that may have acted as confounders, which remains to be confirmed. Lastly, due to the retrospective study design, we did not fully capture the details of chemotherapy and radiotherapy; as an alternative, we focused on one type of cancer. Despite these limitations, the present study fills a gap in the literature and suggests that long-term survivors of CRC should consider abstaining from alcohol consumption to prevent CVD as well as CRC recurrence.

## Conclusion

In this large, retrospective cohort study, no discernable protective association was found between alcohol consumption and incident CVD, not even for light-to-moderate drinking, among long-term survivors of CRC. Alcohol consumption ≥40 g/day in men and ≥ 20 g/day in women was associated with increased CVD, which was attributed mostly to stroke rather than to IHD. Considering that CRC is an alcohol-related cancer and that both its development and mortality are increased by alcohol consumption, long-term survivors with CRC should moderate and, if possible, abstain from drinking alcohol to prevent CVD and CRC recurrence. The results from this study may provide useful evidence when advising survivors of cancer regarding alcohol use.

## Supplementary Information

**Additional file 1 **: **Figure S1**. Study design. **Table S1**. Subgroup analysis on the association of alcohol consumption with the risk of cardiovascular disease among men with long-term colorectal cancer survivors. **Table S2**. Subgroup analysis on association of alcohol consumption with the risk of cardiovascular disease among women with long-term colorectal cancer survivors. **Table S3**. Subgroup analysis on association of alcohol consumption with CVD according to the World Health Organization classification.

## Data Availability

The datasets generated and/or analysed during the current study are available in the Institutional Review Board of Korean National Health Insurance Service repository, https://nhiss.nhis.or.kr.

## Abbreviations

aHR: Adjusted hazard ratio
CCI: Charlson Comorbidity Index
CI: Confidence interval
CRC: Colorectal cancer
CVD: Cardiovascular disease
HR: Hazard ratio
ICD-10: International Classification of Diseases, Tenth Revision
IHD: Ischemic heart disease
NHIS: National Health Insurance Service
WHO: World Health Organization
