# Supplementary material for: Associations between alcohol consumption and cardiovascular disease among long-term survivors of colorectal cancer: a population-based, retrospective cohort study
Source: BMC Cancer. 2021 Jun 16;21:710. doi: 10.1186/s12885-021-08436-w (PMC8207645; doi:10.1186/s12885-021-08436-w)
Supplement: Supplementary file 1 — Additional file 1 : Figure S1. Study design. Table S1. Subgroup analysis on the association of alcohol consumption with the risk of cardiovascular disease among men with long-term colorectal cancer survivors. Table S2. Subgroup analysis on association of alcohol consumption with the risk of cardiovascular disease among women with long-term colorectal cancer survivors. Table S3. Subgroup analysis on association of alcohol consumption with CVD according to the World Health Organization classification. [file 12885_2021_8436_MOESM1_ESM.docx]

**
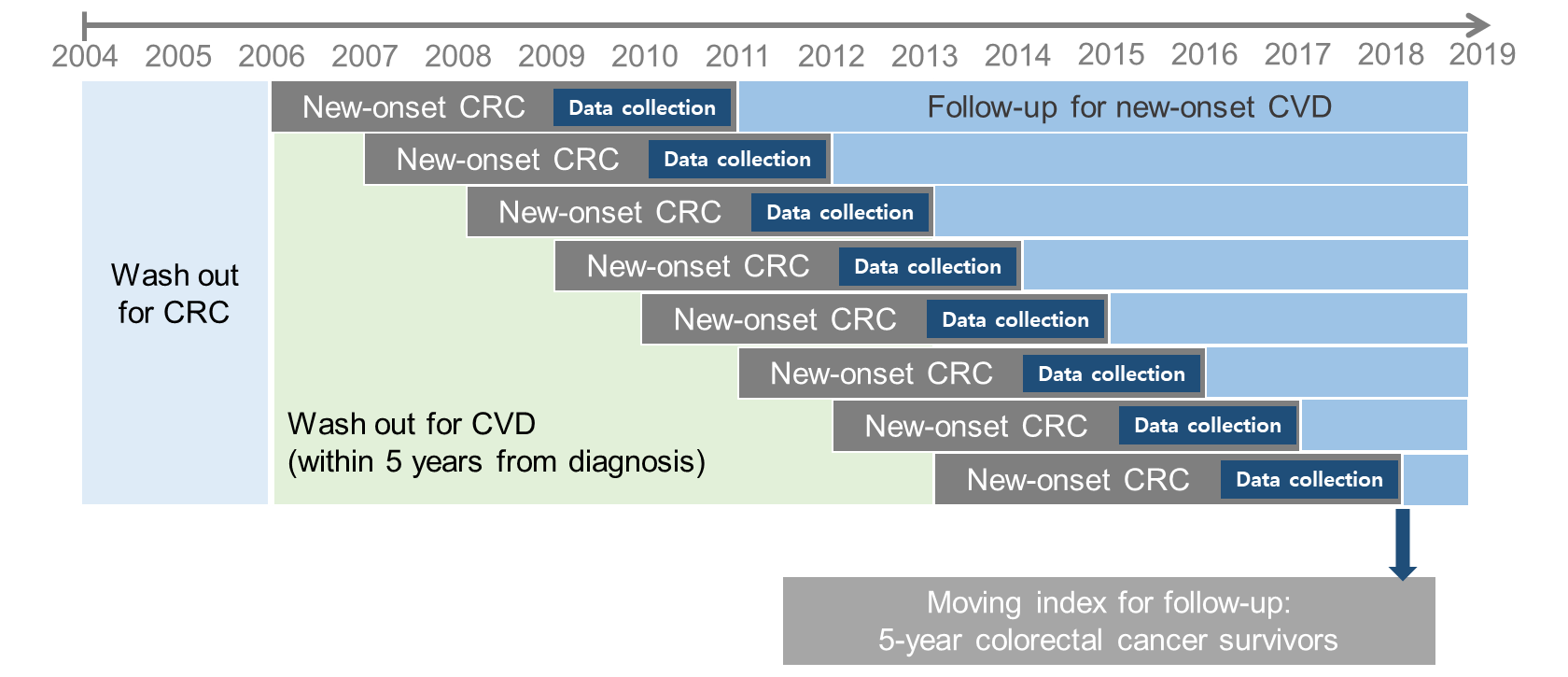
**

**Figure S1. Study design.** All patients with diagnosis of colorectal cancer before 2006 were washed out for colorectal cancer. Data of new-onset colorectal cancer patients were obtained within 2 years before the date of 5-year survival. Follow-up investigation was carried out from the date of 5-year survival to the date of event, death, or 31 December 2018, whichever came earlier.

**Table S1. Subgroup analysis on the association of alcohol consumption with the risk of cardiovascular disease among men with long-term colorectal cancer survivors**

| Outcome | 0 g/day  (n=6,888) | 0.1-9.9 g/day  (n=1,903) | 10-19.9 g/day  (n=1,064) | 20-29.9 g/day  (n=781) | 30-39.9 g/day  (n=350) | ≥40 g/day  (n=788) | *P* for trend |
| --- | --- | --- | --- | --- | --- | --- | --- |
| Age≥65 years | 1.00 (Reference) | 1.10 (0.84-1.43) | 0.91 (0.63-1.31) | 1.17 (0.77-1.78) | 1.34 (0.81-2.24) | 1.26 (0.88-1.79) | 0.587 |
| Age<65 years | 1.00 (Reference) | 0.75 (0.46-1.23) | 0.84 (0.48-1.49) | 1.33 (0.77-2.28) | 0.77 (0.31-1.94) | 1.59 (0.96-2.62) | 0.151 |
| BMI≥25 kg/m^2^ | 1.00 (Reference) | 0.99 (0.65-1.51) | 1.26 (0.78-2.03) | 1.02 (0.56-1.83) | 1.08 (0.50-2.34) | 1.40 (0.85-2.28) | 0.780 |
| BMI<25 kg/m^2^ | 1.00 (Reference) | 1.02 (0.77-1.34) | 0.73 (0.48-1.09) | 1.43 (0.97-2.12) | 1.22 (0.70-2.11) | 1.39 (0.98-1.98) | 0.075 |
| Dyslipidemia | 1.00 (Reference) | 2.91 (0.84-10.1) | 7.47 (1.90-29.4) | 4.35 (1.14-16.7) | 3.56 (0.38-33.5) | 3.12 (0.86-11.4) | 0.070 |
| No dyslipidemia | 1.00 (Reference) | 0.98 (0.77-1.24) | 0.83 (0.60-1.15) | 1.19 (0.85-1.67) | 1.14 (0.72-1.80) | 1.37 (1.02-1.83) | 0.187 |
| Hypertension | 1.00 (Reference) | 1.02 (0.71-1.47) | 0.79 (0.48-1.31) | 1.37 (0.86-2.17) | 1.07 (0.56-2.06) | 1.30 (0.83-2.02) | 0.529 |
| No hypertension | 1.00 (Reference) | 0.97 (0.71-1.31) | 0.91 (0.61-1.35) | 1.05 (0.67-1.67) | 1.18 (0.64-2.17) | 1.41 (0.97-2.05) | 0.513 |
| DM | 1.00 (Reference) | 1.25 (0.77-2.04) | 0.70 (0.33-1.48) | 1.82 (0.96-3.48) | 1.45 (0.51-4.15) | 1.34 (0.71-2.53) | 0.314 |
| No DM | 1.00 (Reference) | 0.95 (0.73-1.23) | 0.95 (0.68-1.34) | 1.15 (0.78-1.68) | 1.15 (0.70-1.89) | 1.45 (1.05-2.00) | 0.259 |
| CCI≥2 | 1.00 (Reference) | 1.02 (0.80-1.31) | 0.90 (0.65-1.26) | 1.35 (0.95-1.91) | 1.30 (0.82-2.06) | 1.26 (0.92-1.74) | 0.295 |
| CCI<2 | 1.00 (Reference) | 0.89 (0.46-1.73) | 0.82 (0.37-1.82) | 0.77 (0.31-1.91) | 0.37 (0.05-2.72) | 2.31 (1.15-4.62) | 0.075 |
| Ever smoker | 1.00 (Reference) | 1.26 (0.95-1.67) | 0.99 (0.69-1.43) | 1.57 (1.09-2.26) | 1.17 (0.69-1.98) | 1.44 (1.02-2.03) | 0.076 |
| Never smoker | 1.00 (Reference) | 0.67 (0.43-1.02) | 0.81 (0.45-1.46) | 0.76 (0.33-1.71) | 1.51 (0.66-3.41) | 1.70 (1.03-2.82) | 0.059 |
| MVPA | 1.00 (Reference) | 1.05 (0.78-1.43) | 1.13 (0.76-1.67) | 1.64 (1.09-2.46) | 1.41 (0.78-2.56) | 1.86 (1.27-2.73) | 0.015 |
| No MVPA | 1.00 (Reference) | 0.98 (0.68-1.40) | 0.67 (0.40-1.10) | 0.85 (0.49-1.49) | 0.94 (0.48-1.85) | 1.02 (0.66-1.57) | 0.731 |
| Walking | 1.00 (Reference) | 1.01 (0.77-1.32) | 0.83 (0.57-1.20) | 1.60 (1.12-2.27) | 1.34 (0.80-2.25) | 1.61 (1.15-2.26) | 0.006 |
| No walking | 1.00 (Reference) | 1.03 (0.65-1.62) | 1.06 (0.62-1.84) | 0.48 (0.19-1.18) | 0.87 (0.35-2.15) | 0.99 (0.58-1.71) | 0.729 |

Acronym: BMI, body mass index; DM, diabetes mellitus; CCI, Charlson comorbidity index; MVPA, moderate-to-vigorous physical activity; PA, physical activity.

Hazard ratio calculated using Cox proportional hazards regression after adjustments for age, household income, BMI, systolic blood pressure, fasting serum glucose, total cholesterol, smoking, MVPA, walking, CCI, history of chemotherapy, and history of radiotherapy.

**Table S2. Subgroup analysis on association of alcohol consumption with the risk of cardiovascular disease among women with long-term colorectal cancer survivors**

| Outcome | 0 g/day  (n=8,084) | 0.1-9.9 g/day  (n=606) | 10-19.9 g/day  (n=95) | ≥20 g/day  (n=94) | *P* for trend |
| --- | --- | --- | --- | --- | --- |
| Age≥65 years | 1.00 (Reference) | 0.69 (0.30-1.56) | 1.32 (0.32-5.40) | 3.65 (1.57-8.51) | 0.017 |
| Age<65 years | 1.00 (Reference) | 0.98 (0.43-2.26) | 1.13 (0.27-4.67) | 4.47 (1.79-11.12) | 0.015 |
| BMI≥25 kg/m^2^ | 1.00 (Reference) | 0.33 (0.08-1.34) | 2.76 (0.86-8.89) | 4.12 (1.48-11.51) | 0.006 |
| BMI<25 kg/m^2^ | 1.00 (Reference) | 1.12 (0.58-2.14) | 0.41 (0.06-3.01) | 3.27 (1.48-7.24) | 0.021 |
| Dyslipidemia | 1.00 (Reference) | NA | 5.43 (0.60-49.25) | 4.63 (1.14-18.88) | 0.109 |
| No dyslipidemia | 1.00 (Reference) | 0.94 (0.52-1.68) | 0.92 (0.29-2.89) | 3.38 (1.65-6.92) | 0.010 |
| Hypertension | 1.00 (Reference) | 1.22 (0.56-2.65) | 1.20 (0.29-4.98) | 3.83 (1.68-8.73) | 0.016 |
| No hypertension | 1.00 (Reference) | 0.57 (0.23-1.39) | 1.00 (0.24-4.10) | 3.07 (1.18-7.94) | 0.065 |
| DM | 1.00 (Reference) | NA | 8.38 (1.62-43.22) | 3.19 (0.42-24.35) | 0.054 |
| No DM | 1.00 (Reference) | 0.94 (0.52-1.69) | 0.65 (0.16-2.64) | 4.00 (2.05-7.80) | <0.001 |
| CCI≥2 | 1.00 (Reference) | 0.86 (0.48-1.53) | 1.18 (0.43-3.19) | 3.31 (1.75-6.25) | 0.003 |
| CCI<2 | 1.00 (Reference) | NA | NA | 53.45 (3.58-797.50) | 0.040 |
| Ever smoker | 1.00 (Reference) | NA | 2.27 (0.21-24.14) | 2.90 (0.41-20.81) | 0.727 |
| Never smoker | 1.00 (Reference) | 0.89 (0.50-1.59) | 1.08 (0.34-3.38) | 4.60 (2.43-8.69) | <0.001 |
| MVPA | 1.00 (Reference) | 0.41 (0.13-1.30) | 0.55 (0.08-3.97) | 2.81 (0.99-7.93) | 0.078 |
| No MVPA | 1.00 (Reference) | 1.16 (0.59-2.29) | 1.62 (0.51-5.17) | 4.24 (1.97-9.14) | 0.003 |
| Walking | 1.00 (Reference) | 0.70 (0.34-1.43) | 0.74 (0.18-3.02) | 2.29 (0.98-5.36) | 0.167 |
| No walking | 1.00 (Reference) | 1.16 (0.42-3.21) | 2.34 (0.55-10.04) | 9.17 (3.61-23.28) | <0.001 |

Acronym: BMI, body mass index; NA, not applicable; DM, diabetes mellitus; CCI, Charlson comorbidity index; MVPA, moderate-to-vigorous physical activity; PA, physical activity.

Hazard ratio calculated using Cox proportional hazards regression after adjustments for age, household income, BMI, systolic blood pressure, fasting serum glucose, total cholesterol, smoking, MVPA, walking, CCI, history of chemotherapy, and history of radiotherapy.

**Table S3. Subgroup analysis on association of alcohol consumption with CVD according to the World Health Organization classification**

| Subgroup | No drinking  (n=14,972) | Moderate drinking (n=4,779) | Hazardous drinking  (n=882) | *P* for trend |
| --- | --- | --- | --- | --- |
| Male | 1.00 (Reference) | 1.03 (0.86-1.23) | 1.39 (1.04-1.85) | 0.074 |
| Female | 1.00 (Reference) | 1.03 (0.65-1.63) | 3.37 (1.61-7.04) | 0.006 |
| Age≥65 years | 1.00 (Reference) | 1.11 (0.91-1.34) | 1.35 (0.96-1.89) | 0.179 |
| Age<65 years | 1.00 (Reference) | 0.95 (0.69-1.31) | 1.77 (1.13-2.78) | 0.020 |
| Body mass index≥25 kg/m^2^ | 1.00 (Reference) | 1.09 (0.81-1.45) | 1.57 (1.00-2.48) | 0.148 |
| Body mass index<25 kg/m^2^ | 1.00 (Reference) | 1.06 (0.86-1.29) | 1.50 (1.08-2.08) | 0.055 |
| Dyslipidemia | 1.00 (Reference) | 1.97 (1.04-3.74) | 1.44 (0.47-4.44) | 0.115 |
| No dyslipidemia | 1.00 (Reference) | 1.02 (0.85-1.21) | 1.51 (1.14-1.98) | 0.012 |
| Hypertension | 1.00 (Reference) | 1.12 (0.87-1.44) | 1.43 (0.95-2.16) | 0.212 |
| No hypertension | 1.00 (Reference) | 0.98 (0.79-1.22) | 1.52 (1.07-2.15) | 0.050 |
| Diabetes mellitus | 1.00 (Reference) | 1.15 (0.79-1.66) | 1.28 (0.70-2.34) | 0.630 |
| No diabetes mellitus | 1.00 (Reference) | 1.04 (0.87-1.26) | 1.60 (1.19-2.16) | 0.008 |
| CCI≥2 | 1.00 (Reference) | 1.09 (0.92-1.30) | 1.37 (1.02-1.83) | 0.103 |
| CCI<2 | 1.00 (Reference) | 0.80 (0.47-1.33) | 2.57 (1.31-5.03) | 0.002 |
| Ever smoker | 1.00 (Reference) | 1.21 (0.98-1.51) | 1.45 (1.04-2.03) | 0.053 |
| Never smoker | 1.00 (Reference) | 0.89 (0.68-1.16) | 2.00 (1.30-3.08) | 0.003 |
| MVPA | 1.00 (Reference) | 1.14 (0.91-1.43) | 1.81 (1.25-2.61) | 0.007 |
| No MVPA | 1.00 (Reference) | 0.96 (0.75-1.23) | 1.27 (0.86-1.87) | 0.413 |
| Walking | 1.00 (Reference) | 1.05 (0.86-1.27) | 1.63 (1.19-2.23) | 0.010 |
| No walking | 1.00 (Reference) | 1.09 (0.79-1.50) | 1.24 (0.75-2.03) | 0.670 |

Data are aHR (95% CI). HR calculated using Cox proportional hazards regression after adjustments for age, sex, household income, systolic blood pressure, body mass index, fasting serum glucose, total cholesterol, smoking, physical activity, Charlson comorbidity index, history of chemotherapy, and history of radiotherapy.

Acronym: HR, hazard ratio; CI, confidence interval; CCI, Charlson comorbidity index; MVPA, moderate-to-vigorous physical activity.
